# Phytogeographical regions of Egypt: first open-source geospatial data and its applications

**DOI:** 10.1186/s13104-023-06528-3

**Published:** 2023-10-09

**Authors:** Amr E. Keshta

**Affiliations:** Botany Department, College of Science, Tanta University, Tanta, 31512 Egypt

**Keywords:** GIS, Biodiversity, Digitization, Nile River, Sinai

## Abstract

**Objectives:**

To our knowledge, this is the first attempt for digitizing the Egyptian phytogeographical regions through incorporation of Geographical Information Systems (GIS) techniques including geo-referencing ground data of old-history paper maps. The main objective for the current study was digitizing and creating the first open-source geospatial data for the Egyptian phytogeographical regions and to make them readily available for usage by researchers - making this study novel.

**Data description:**

Geospatial data were created for the Egyptian phytogeographical regions based on ground data paper map showing the boundaries of each region in the country, Egypt. Digitization of the boundaries of each region was executed using ArcMap 10.4 followed by quality checks executions for ensuring the quality and accuracy of the created geospatial data. The data created in this study are available as file geodatabase (.gdb) and shapefile. Having the Egyptian phytogeographical regions available for GIS analysts and cartographers as geospatial data is a powerful tool for further research applications including phytoremediation, biodiversity, conservation, GIS, and remote sensing studies.

## Objective

Plant research studies and their distribution in relation to phytogeographical regions in Egypt of a great interest to scientist and continue to be a vital topic for interdisciplinary research among ecologist, geographers, and geospatial scientist. Research scientists classified Egypt into several phytogeographical regions based on plant species and their influence on the surrounding environment [1–3]. El Hadidi [1] classified Egypt into Nile region (sub classified into Nile delta (Nd), Nile valley (Nv), and Nile Faiyum (Nf)), Oases of the western desert region (O), Mediterranean region (sub classified into Western Mediterranean coastal region (Mm) and Eastern Mediterranean coastal region (Ms), and Desert region (sub classified into Eastern desert (Da sept), Isthmic desert (Di), Western desert (Dl)), Ge: Gebel Elba, Red Sea (R), and Sinai (S), .

GIS has many applications in biodiversity monitoring, species distribution, and assessing habitat loss [4–10] – implying evolving and emerging needs for open-source and free geospatial data. Studying phytogeography is vital for assessing the spatial variability in plant distribution and floristic composition in a given study area. Moreover, research and studies based on phytogeography are vital in understanding species migration, origination, speciation, and conservation [11–14]. For the last few decades, phytogeographical regions of Egypt, especially Nile Delta, were severely impacted by human activities [15] including cultivation for Wadi deltaic regions, medicinal plant harvesting due to their economic and medical values, new cities creation, and road establishment [15–18]. Such anthropogenic activities impacted the natural flora and changed geographical distribution of plant species [16–22].

## Data description

Table 1 provides an overview of the data files and datasets stored in Figshare. Data file 1 shows a map for the digitized Egyptian phytogeographical regions [23], while data set 1 hold the created geospatial data [24] for the Egyptian phytogeographical regions as a shapefile and a file geodatabase. Data file 2 shows a flow chart for the adopted methodology of geospatial data creation [25]. For creating the geospatial data for the phytogeographical regions of Egypt, the following procedures [26, 27] were executed:

1. Creating file geodatabase (FGdb)
  A file geodatabase (FGdb) was created on a local drive using ArcCatalog 10.4 where all geospatial data stored.
2. Ground data paper map originally produced by El Hadidi [1] showing the boundaries of each phytogeographical region were scanned and saved as a Tag Image File Format (TIFF). Consequently, georeferencing protocol [27] was executed (Total georeferencing RMS error was 9.8 m). In regard to mapping accuracy, standard and well defined thematic [28] and geometric accuracy [29] methods were executed to ensure overall mapping accuracy – including error matrix. All vector data were spatially projected and georeferenced to the World Geodetic System (WGS 1984) datum. All GIS workflow was executed using ArcGIS Desktop 10.4.
3. Creating a polygon feature class
  A polygon feature class was created inside the FGdb to be used while digitization and creation of vertices of the phytogeographical boundaries.
4. Digitization and creating attribute table
  Shapefile of Egypt (as a polygon feature class) was used to identify the outside borders of the country and the georeferenced TIFF image of the phytogeographical were loaded up. Digitization process were executed for creating the vertices of each class of the phytogeographical regions. Attribute table was created along the polygon feature class where all the field names (e.g. phytogeographical name) were populated and filled accordingly.
5. Quality Assurance and Quality Control (QA/QC) workflow
  Upon digitization completion, a QA/QC workflow was executed to ensure the quality and accuracy of the created geospatial data. QA/QC Data Reviewer checks that were executed include invalid geometry, multipart polygon, polygon silver, and no overlap checks using Data Reviewer ArcGIS 10.4 Extension. Executing QA/QC checks returns no errors, except the overlap checks that revealed overlap between some of the regions. All overlap issues were resolved and the polygon overlap check executed again and returns no errors. Moreover, metadata were completed according to the guidelines of the Federal Geographic Data Committee’s (FGDC) Content Standard for Digital Spatial Metadata (CSDGM).

**Table 1:** Overview of data files/data sets

Label	Name of data file/data set	File types (file extension)	Data repository and identifier
Data file 1	Phytogeographical regions of Egypt	Image (.JPEG)	Figshare (10.6084/m9.figshare.21368982.v1) (23)
Data set 1	GIS data of phytogeographical regions of Egypt	File geodatabase (.gdb)	Figshare (10.6084/m9.figshare.21368928.v1) (24)
Data file 2	Flow chart of adopted methodology of geospatial data creation	Image (.JPEG)	Figshare (10.6084/m9.figshare.24041943.v1) (25)

## Limitations

The geospatial data digitized and created in the current study is limited to the ground data [1] paper map only based on which the geospatial digitization executed. The mapping accuracy for the generated geospatial data is limited to and based on the topographic objects identified in the ground data paper map available during the current study. Moreover, many factors can impact the phytogeographical classification of a particular study area including flora, climate, and geography [30]. Geospatial data are vital for many applications including predicting geographic patterns, assessing land changes, and providing insights about potential effects. Accordingly, it is highly recommended for creating and applying more geospatial data for developing countries like Egypt and make that data readily accessible to researchers, scientists, urban planners, GIS analysts…. etc. for a more sustainable environment that could cope with ongoing climate change.

## Data Availability

The data described in this Data note are freely and openly available on the Figshare repository at 10.6084/m9.figshare.21368928.v1. Anyone can access the data at Figshare website searching with keywords: Geospatial data, phytogeographical regions, and Egypt. Please see Table 1 and references [23–25] for details and links to the data.
